# N-truncated Aβ_4–x_ peptides in sporadic Alzheimer’s disease cases and transgenic Alzheimer mouse models

**DOI:** 10.1186/s13195-017-0309-z

**Published:** 2017-10-04

**Authors:** Oliver Wirths, Susanne Walter, Inga Kraus, Hans W. Klafki, Martina Stazi, Timo J. Oberstein, Jorge Ghiso, Jens Wiltfang, Thomas A. Bayer, Sascha Weggen

**Affiliations:** 1 Division of Molecular Psychiatry, University Medical Center (UMG), Georg-August-University, von-Siebold-Strasse 5, 37075 Goettingen, Germany; 2 Department of Psychiatry and Psychotherapy, University Medical Center (UMG), Georg-August-University, von-Siebold-Strasse 5, 37075 Goettingen, Germany; 3 Department of Neuropathology, Heinrich-Heine-University, Düsseldorf, Germany; 4 Department of Psychiatry and Psychotherapy, Friedrich-Alexander-University of Erlangen-Nuremberg, Erlangen, Germany; 5 Department of Pathology, New York University School of Medicine, New York, NY USA; 6 Departments of Psychiatry, New York University School of Medicine, New York, NY USA; 7 German Center for Neurodegenerative Diseases (DZNE), Göttingen, Germany; 8 Institute for Research in Biomedicine (iBiMED), Medical Sciences Department, University of Aveiro, Aveiro, Portugal

**Keywords:** Alzheimer’s disease, N-truncated Amyloid-β, Immunohistochemistry, Mouse model, Postmortem, 5XFAD, APP/PS1KI

## Abstract

**Background:**

The deposition of neurotoxic amyloid-β (Aβ) peptides in plaques in the brain parenchyma and in cerebral blood vessels is considered to be a key event in Alzheimer’s disease (AD) pathogenesis. Although the presence and impact of full-length Aβ peptides such as Aβ_1–40_ and Aβ_1–42_ have been analyzed extensively, the deposition of N-terminally truncated Aβ peptide species has received much less attention, largely because of the lack of specific antibodies.

**Methods:**

This paper describes the generation and characterization of novel antibodies selective for Aβ_4–x_ peptides and provides immunohistochemical evidence of Aβ_4–x_ in the human brain and its distribution in the APP/PS1KI and 5XFAD transgenic mouse models.

**Results:**

The Aβ_4–x_ staining pattern was restricted mainly to amyloid plaque cores and cerebral amyloid angiopathy in AD and Down syndrome cases and in both AD mouse models. In contrast, diffuse amyloid deposits were largely negative for Aβ_4–x_ immunoreactivity. No overt intraneuronal staining was observed.

**Conclusions:**

The findings of this study are consistent with previous reports demonstrating a high aggregation propensity of Aβ_4–x_ peptides and suggest an important role of these N-truncated Aβ species in the process of amyloidogenesis and plaque core formation.

**Electronic supplementary material:**

The online version of this article (doi:10.1186/s13195-017-0309-z) contains supplementary material, which is available to authorized users.

## Background

Alzheimer’s disease (AD) is a severe age-dependent neurodegenerative disorder accounting for the majority of dementia cases worldwide. It is characterized by two neuropathological hallmark lesions, namely the accumulation of amyloid-β (Aβ) peptides in the form of extracellular plaques and the formation of intracellular neurofibrillary tangles consisting of hyperphosphorylated tau protein [1]. The formation of extracellular plaques is mechanistically linked to the amyloid cascade hypothesis, positioning the accumulation of Aβ peptides as a pivotal and triggering event in AD etiology [2].

The production of Aβ peptides depends on the sequential processing of the type I single transmembrane amyloid precursor protein (APP) by two proteases called β- and γ-secretase [3]. In addition to full-length Aβ_1–40_ and Aβ_1–42_ peptides, a variety of peptides with shorter C-termini, such as Aβ_1–37_, Aβ_1–38_, or Aβ_1–39_, have been described [4, 5]. Furthermore, elongated amyloidogenic Aβ_1–43_ peptides have recently been reported [6]. Similar heterogeneity has been observed at the Aβ N-terminus, and several N-terminally truncated Aβ forms, including Aβ_2–x_ [7], Aβ_pE3–x_ [8, 9], Aβ_5–x_ [10, 11], and Aβ_pE11–x_ [9, 12, 13], have been demonstrated in parenchymal or vascular deposits of human AD brains by immunohistochemical methods. An even more complex pattern of Aβ peptides has been reported by the use of mass spectrometry [14–17], which detected further variants such as Aβ_7–42_, Aβ_8–42_, or Aβ_9–42_. N-terminal truncations are believed to render Aβ peptides more neurotoxic and to promote enhanced peptide aggregation compared with the corresponding full-length peptides with an intact N-terminus [18].

Aβ_4–x_ peptides appear to be among the most abundant Aβ species, but not much is known about the spatial distribution of these N-terminally truncated peptides in AD brains. Already in 1985, Masters and colleagues [19] reported the presence of a major Aβ peptide species starting with the phenylalanine (Phe) at position 4 of the Aβ sequence in both AD and Down syndrome (DS) cases, an observation that was subsequently verified [20]. More recent mass spectrometric characterizations of Aβ isoform signatures in human brain samples confirmed that Aβ_4–42_ and Aβ_1–42_ represent highly abundant species in cortical samples of AD and control brains [14, 21]. Whether Aβ_4–x_ peptides also occur within intracellular compartments is currently unclear. Likewise, the origin of Aβ_4–x_ peptides remains unknown. In this respect, a recent in vitro study suggested that neurons predominantly secrete full-length Aβ peptides starting with the aspartate in position 1, whereas astrocytes and microglia in addition secrete measurable amounts of N-terminally truncated species, including Aβ_4/5–x_ [22]. The aim of the present work was to characterize newly generated Aβ_4–x_-specific antibodies as a tool to study Aβ_4–x_ peptides in different AD transgenic mouse models and human patients with sporadic AD.

## Methods

### Development of Aβ_4–x_-specific polyclonal antibodies in guinea pigs

Antibodies were generated by the Peptide Specialty Laboratory (PSL; Heidelberg, Germany). In brief, immunization was carried out in two guinea pigs using the six-amino acid peptide (FRHDSG) corresponding to residues (4–9) of the Aβ peptide and coupled at its C-terminus via an additional cysteine residue to maleimide-activated keyhole limpet hemocyanin (KLH) (Thermo Fisher Scientific, Waltham, MA, USA). Antisera were generated by injection of peptides conjugated to KLH and emulsified with complete Freund’s adjuvant for a first injection, followed by injections 2, 3, and 4 at 2-week intervals with incomplete Freund’s adjuvant. Serum was collected prior to the first immunization (preimmune serum) and after the fourth immunization. The immune sera obtained after the final bleed were affinity-purified and named 029-1 and 029-2.

### Antibody characterization by capillary isoelectric focusing immunoassay and Western immunoblot analysis

To assess the selectivity of purified polyclonal antibodies 029-1 and 029-2 for different N-terminal variants of Aβ, we employed a capillary isoelectric focusing (CIEF) immunoassay [23] and urea-bicine/bis-tris/Tris/sulfate sodium dodecyl sulfate (SDS)-PAGE followed by Western immunoblotting [22, 24]. For the CIEF immunoassay, synthetic Aβ peptides with different N-termini were separated on a Peggy Sue device (ProteinSimple, San Jose, CA, USA) by isoelectric focusing in microcapillaries and subsequently were probed with purified 029-1 and 029-2 antibodies. For comparison, the CIEF immunoassay was also performed with the mouse monoclonal antibodies IC16 [25] and 6E10 (BioLegend, San Diego, CA, USA), which recognize several N-terminal variants of Aβ. The synthetic Aβ peptides Aβ_1–40_, Aβ_2–40_, Aβ_3–40_, pyroglutamate Aβ_pE3–40_, Aβ_4–40_, and Aβ_5–40_ were purchased from AnaSpec (Fremont, CA, USA). Peptide stock solutions (1 mg/ml) were prepared in dimethyl sulfoxide (DMSO) or, in the case of Aβ_pE3–40_, in 0.1% NH_4_OH and stored at −80 °C. Aliquots were thawed only once. The peptides were further diluted in 20 mM bicine, pH 7.6, with 0.6% 3-[(3-cholamidopropyl)dimethylammonio]-1-propanesulfonate hydrate and then mixed 1:4 with Premix G2 containing a pH 5–8 nested gradient, fluorescent pH standards, and a DMSO inhibitor mix (all reagents were obtained from ProteinSimple). The automated CIEF immunoassay on the Peggy Sue platform (ProteinSimple) was carried out as described previously for the NanoPro platform (ProteinSimple) [23]. The final peptide concentration in the microcapillaries was 100 ng/ml per peptide. Assuming an internal capillary volume of roughly 0.5 μl, this corresponded to approximately 50 pg of peptide per capillary. Primary antibodies 029-1, 029-2, 6E10, and IC16 were diluted in antibody diluent (ProteinSimple). Detection was achieved with biotinylated anti-guinea pig immunoglobulin G (IgG; Dianova, Barcelona, Spain) in combination with streptavidin-horseradish peroxidase (HRP) (ProteinSimple) for antibodies 029-1 and 029-2 or with goat-anti-mouse-HRP secondary antibody (ProteinSimple) for 6E10 and IC16.

For SDS-PAGE analysis, stock solutions of synthetic Aβ_1–40_, Aβ_2–40_, Aβ_3–40_, pyroglutamate Aβ_3–40_ (Aβ_pE3–40_), and Aβ_4–40_ were prepared in sample buffer (0.36 M bis-tris, 0.16 M bicine, 15% wt/vol sucrose, 1% wt/vol SDS, and 0.0075% wt/vol bromophenol blue) and stored at −80 °C. The Aβ peptides were further diluted in sample buffer, and aliquots containing 0.1, 1, and 10 ng of peptide per lane were separated by urea-bicine/bis-tris/Tris/sulfate SDS-PAGE and analyzed by immunoblotting as previously described [7]. The blotting membranes were blocked in 2% electrochemiluminescence (ECL) advance blocking agent (GE Healthcare Life Sciences, Little Chalfont, UK) in PBS with 0.1% Tween 20 (PBS-T) for at least 4 h at room temperature (RT) and probed with the primary antibody 029-2 (1:300 in PBS-T) at 4 °C overnight. After three washing steps with PBS-T, biotinylated goat anti-guinea pig IgG (1:3000 in PBS-T; Life Technologies, Carlsbad, CA, USA) and streptavidin-HRP were employed as secondary reagents. Chemiluminescent signals obtained with ECL Prime Western Blotting Detection Reagent (GE Healthcare Life Sciences) were recorded. After three additional washing steps in PBS-T, the membrane was reprobed with 6E10 (1:1000 in PBS-T) and developed with biotinylated goat anti-mouse IgG (333 ng/ml in PBS-T; Vector Laboratories Ltd., Peterborough, UK) in combination with streptavidin-HRP complex (1:3000 in PBS-T).

### Peptide competition assay

To further assess the selectivity of the Aβ_4–x_-specific polyclonal antibodies, a peptide competition experiment was performed. Antibody solution 029-2 was incubated with an excess of synthetic Aβ_1–40_ or Aβ_4–40_ for 5 h at RT with constant agitation, followed by centrifugation at 14,000 rpm for 5 minutes. The supernatant was used for immunohistochemical analysis of transgenic 5XFAD mouse brain tissues.

### Human brain samples

Human brain samples from sporadic AD (*n* = 16, mean age 84.18 ± 8.04 years), nondemented control subjects (*n* = 6, mean age 84.17 ± 5.19 years), and DS (*n* = 2, mean age 61.0 ± 4.24 years) were obtained from the Netherlands Brain Bank (Table 1). The present study was approved by the ethics committee of the University Medical Center Göttingen.

**Table 1:** Clinical and pathological data of sporadic Alzheimer’s disease cases, Down syndrome cases, and nondemented control subjects

				Aβ_4–x_ (029-2)		Aβ (IC16)	
Case	Age (years)	Braak stage	ApoE	SP	CAA	SP	CAA
Sporadic AD cases							
AD-1	87	V	3/3	++	++	+++	++
AD-2	83	VI	4/3	+	+	+++	+
AD-3	78	V	4/3	++	+	+++	++
AD-4	79	IV	4/3	++	+++	++	+++
AD-5	82	V	4/4	+	+	+++	++
AD-6	84	IV	4/3	++	++	+++	+++
AD-7	85	VI	3/3	(+)	−	+++	++
AD-8	92	IV	3/3	+	+	++	+
AD-9	92	IV	4/2	+	++	+++	++
AD-10	73	IV	4/4	+	++	+++	+++
AD-11	92	IV	3/3	++	+	+++	++
AD-12	93	IV	3/3	+++	(+)	+++	(+)
AD-13	81	IV	n.d.	++	++	+++	+++
AD-14	95	IV	3/3	+	−	++	−
AD-15	91	IV	4/3	+	+	+++	+
AD-16	65	VI	3/3	(+)	−	++	−
AD-17	79	V	3/3	+	+	++	+
Nondemented control cases							
NDC-1	87	II	n.d.	−	−	−	−
NDC-2	90	I	2/2	+	+	++	++
NDC-3	79	II	4/3	+	−	++	−
NDC-4	89	III	n.d.	−	+	−	+
NDC-5	78	I	3/3	−	+	(−)	−
NDC-6	82	I	3/3	−	−	−	−
Down syndrome							
DS-1	64	V	3/3	++	++	+++	+++
DS-2	58	VI	4/3	++	+++	+++	+++

*Abbreviations: Aβ* Amyloid-β, *AD* Alzheimer’s disease, *ApoE* Apolipoprotein E, *CAA* Cerebral amyloid angiopathy, *DS* Down syndrome, *NDC* Nondemented control subject, *SP* Senile plaques

The following semiquantitative scoring criteria were used: − no staining, (+) barely detectable staining, + weak staining, ++ moderate staining, +++ extensive staining

### Alzheimer mouse models

The generation of APP/PS1KI mice has been described previously [26]. In this AD mouse model, human mutant APP751 containing the Swedish and London mutations is overexpressed under the control of the murine Thy-1 promoter, whereas murine presenilin-1 (PSEN1) with the M233T and L235P familial Alzheimer’s disease (FAD)-linked mutations is expressed under the control of the endogenous mouse PSEN1 promoter. APP/PS1KI mice were a generous gift of Dr. Laurent Pradier, Sanofi-Aventis, Paris, France. The analysis of 5XFAD mice (Tg6799) has also been reported previously [27, 28]. 5XFAD mice overexpress APP695 carrying the Swedish, Florida, and London mutations under the control of the murine Thy-1 promoter. In addition, human PS1 carrying the M146L and L286V mutations is also expressed under the control of the murine Thy-1 promoter. Male mice on a C57BL/6 × SJL genetic background were obtained from The Jackson Laboratory [strain B6SJL-Tg(AβPPSwFlLon,PSEN1*M146L*L286V)6799Vas/J; The Jackson Laboratory, Bar Harbor, ME, USA] and backcrossed for ten generations to C57BL/6 J wild-type mice to obtain an incipient congenic line on a C57BL/6 J genetic background. All animals were handled according to German guidelines for animal care.

### Immunohistochemical and immunofluorescence analyses

Mice were killed by CO_2_ anesthetization followed by cervical dislocation. Brain samples were dissected and postfixed in 4% phosphate-buffered formalin at 4 °C. Immunohistochemistry was performed on 4-μm paraffin sections. The antibodies 029-1, 029-2 (1:250–500), D3E10 (against Aβ42, 1:1000; Cell Signaling Technology, Danvers, MA, USA), and IC16 (against the N-terminus of Aβ, 1:1000) were used for Aβ staining. Biotinylated secondary anti-guinea pig and anti-mouse antibodies (1:200) were purchased from Dianova and Dako (Glostrup, Denmark), respectively. Staining was visualized using the avidin-biotin complex method with a VECTASTAIN kit (Vector Laboratories) and diaminobenzidine (DAB) as the chromogen. Counterstaining was carried out with hematoxylin.

The Aβ_4–x_ plaque load was quantified using 9-month-old hemi- and homozygous 5XFAD mice (*n* = 3–4) [28]. In brief, three paraffin sections per animal were stained simultaneously with DAB. The relative Aβ_4–x_ load in thalamus and cortex was evaluated using a BX51 microscope (Olympus, Center Valley, PA, USA) equipped with a Moticam Pro 282 camera (Motic, Wetzlar, Germany) and ImageJ1.47b software (http://imagej.nih.gov/ij). Representative images were captured and binarized to 8-bit black-and-white images using a fixed intensity threshold. Measurements were performed with analysis of the percentage of the area covered by DAB as previously described [27]. Double-immunofluorescence staining was visualized using Alexa Fluor 488- and Alexa Fluor 594-conjugated secondary antibodies (Molecular Probes, Eugene, OR, USA) and 4′,6-diamidino-2-phenylindole using a Nikon C2+ confocal microscope equipped with 405 nm, 488-nm and 561-nm lasers (Nikon Instruments, Melville, NY, USA).

### Enzyme-linked immunosorbent assay

The levels of Aβ_4–40_ peptides in 5-month-old heterozygous (*n* = 5) and homozygous (*n* = 6) 5XFAD mice were determined using a sandwich enzyme-linked immunosorbent assay (ELISA). Frozen brain hemispheres were weighed and sequentially extracted. First, brains were homogenized in 700 μl of Tris-buffered saline (120 mM NaCl, 50 mM Tris, pH 8.0, with cOmplete protease inhibitor cocktail (Roche Diagnostics, Indianapolis, IN, USA) per 100 mg of tissue using a Dounce homogenizer (800 rpm). The resulting solution was centrifuged at 17,000 × *g* for 20 minutes at 4 °C. The pellet was dissolved in 800 μl of 2% SDS and sonicated, followed by a centrifugation step of 17,000 × *g* for 20 minutes at 4 °C. The supernatant containing SDS-soluble proteins was incubated while rotating with 1 μl of benzonase at RT for 10 minutes, followed by storage at −80 °C. The SDS brain fraction was used for ELISA analysis. To generate standard curves, synthetic Aβ_4–40_ peptides (PSL) were used. A C-terminal-specific antibody for Aβ_40_ (BAP-24) [29] served as a capture antibody and was incubated at 3 μg/ml in PBS, pH 7.2, on 96-well high-binding microtiter plates (Greiner Bio-One, Kremsmünster, Austria) overnight at 4 °C. After excess capture antibody was removed, freshly diluted brain samples (1:30) or Aβ peptide standards (31.25–750 pg/ml) in PBS containing 0.05% Tween 20 and 1% bovine serum albumin (BSA) were added. Then, the Aβ_4–x_-specific 029-2 antibody labeled with HRP using the Pierce EZ-LinkTM Plus Activated Peroxidase kit (Thermo Fisher Scientific) was diluted in PBS containing 0.05% Tween 20 and 1% BSA, added to each well, and incubated overnight at 4 °C. On the next day, plates were washed three times with PBS containing 0.05% Tween 20 and once with PBS. Subsequently, 50 μl of trimethylbenzidine ELISA peroxidase substrate (Interchim, Montluçon, France) was added and incubated for 1–5 minutes at RT in the dark. The reaction was terminated by adding 50 μl of 2 M H_2_SO_4_, and the absorbance was recorded using a Paradigm microplate reader (Beckman Coulter, Brea, CA, USA) at 450 nm.

### Statistical analysis

Differences between groups were tested by unpaired *t* tests. All data were expressed as mean ± SD. Significance levels were as follows: ***p < 0.001; **p < 0.01; *p < 0.05. All calculations were performed using Prism version 6.07 for Windows software (GraphPad Software, La Jolla, CA, USA).

## Results

### Purified polyclonal antibodies 029-1 and 029-2 show high selectivity for N-truncated Aβ_4–x_ peptides

**Fig. 1:**
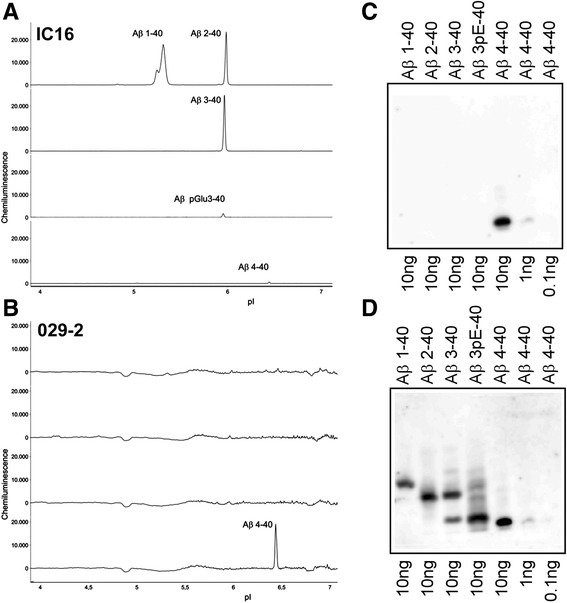
Antibody 029-2 shows high selectivity for amyloid-β (Aβ) peptides starting with the phenylalanine residue in position 4. Synthetic Aβ peptides with different N-termini were analyzed by capillary isoelectric focusing immunoassay (**a**, **b**) or by urea-bicine/bis-tris/Tris/sulfate sodium dodecyl sulfate (SDS)-PAGE followed by Western blotting (**c**, **d**). Mixtures of Aβ_1–40_, Aβ_2–40_, and Aβ_5–40_ (top electropherogram), Aβ_3–40_ (second electropherogram), Aβ_pE3–40_ (third electropherogram), and Aβ_4–40_ (bottom electropherogram) were subjected to isoelectric focusing in microcapillaries and probed with antibody IC16 (**a**) or 029-2 (**b**). Note that the electropherograms shown were derived from different experiments. A set of synthetic Aβ peptides was separated by urea-bicine/bis-tris/Tris/sulfate SDS-PAGE, blotted onto polyvinylidene difluoride membranes and probed with 029-2 (**c**). After a washing step, the same blot was reprobed with 6E10 (**d**). With both methods, 029-2 exclusively detected Aβ_4–x_ peptides, whereas the control antibodies also recognized Aβ peptides with other N-termini

**Fig. 2:**
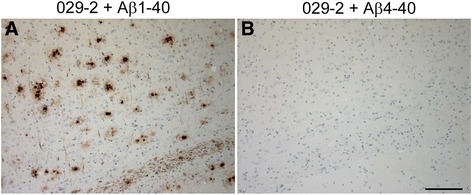
Whereas preabsorption with synthetic amyloid-β (Aβ)_1–40_ did not compromise 029-2 staining in aged 5XFAD mice (**a**), preabsorption with Aβ_4–40_ completely abolished 029-2 immunoreactivity (**b**), underscoring the specificity of the 029-2 antibody for Aβ_4–x_ peptides. Scale bar = 100 μm

The selectivity of the affinity-purified polyclonal antibodies 029-1 and 029-2 for different N-terminal variants of Aβ was studied by three independent methods. First, a CIEF immunoassay demonstrated that both antibodies detected synthetic Aβ peptides starting with the Phe residue in position 4 without any noticeable cross-reactivity to other tested Aβ peptides (Fig. 1b and Additional file 1). In contrast, control antibody IC16 detected the N-terminus of Aβ with clear signals for Aβ_1–40_, Aβ_2–40_, and Aβ_3–40_. Reactivity to Aβ_pE3–40_ and Aβ_4–40_ was very low, whereas Aβ_5–40_ was not detected (Fig. 1a). Antibody 6E10 served as an additional control and detected all synthetic peptides applied (Additional file 1: Figure S1) as previously shown [7]. Second, urea bicine/bis-tris/Tris/sulfate SDS-PAGE followed by Western immunoblotting was performed for 029-2 and indicated that this antibody selectively recognized Aβ peptides starting with Phe in position 4 in amounts as low as 1 ng. Again, no cross-reactivity was detected against any of the other synthetic Aβ peptides tested, namely Aβ_1–40_, Aβ_2–40_, Aβ_3–40_, and Aβ_pE3–40_ (Fig. 1c). Reprobing of the membrane with antibody 6E10 allowed the detection of all tested Aβ peptides (Fig. 1d). Third, importantly, preabsorption of 029-2 with synthetic Aβ_4–40_ peptides completely blocked 029-2 immunoreactivity in formalin-fixed, paraffin-embedded brain tissue from 5XFAD mice. In contrast, preabsorption with full-length Aβ_1–40_ peptides did not result in reduced immunoreactivity, and Aβ_4–x_-positive extracellular deposits were still detected (Fig. 2).

### Aβ_4–x_ in sporadic AD cases

**Fig. 3:**
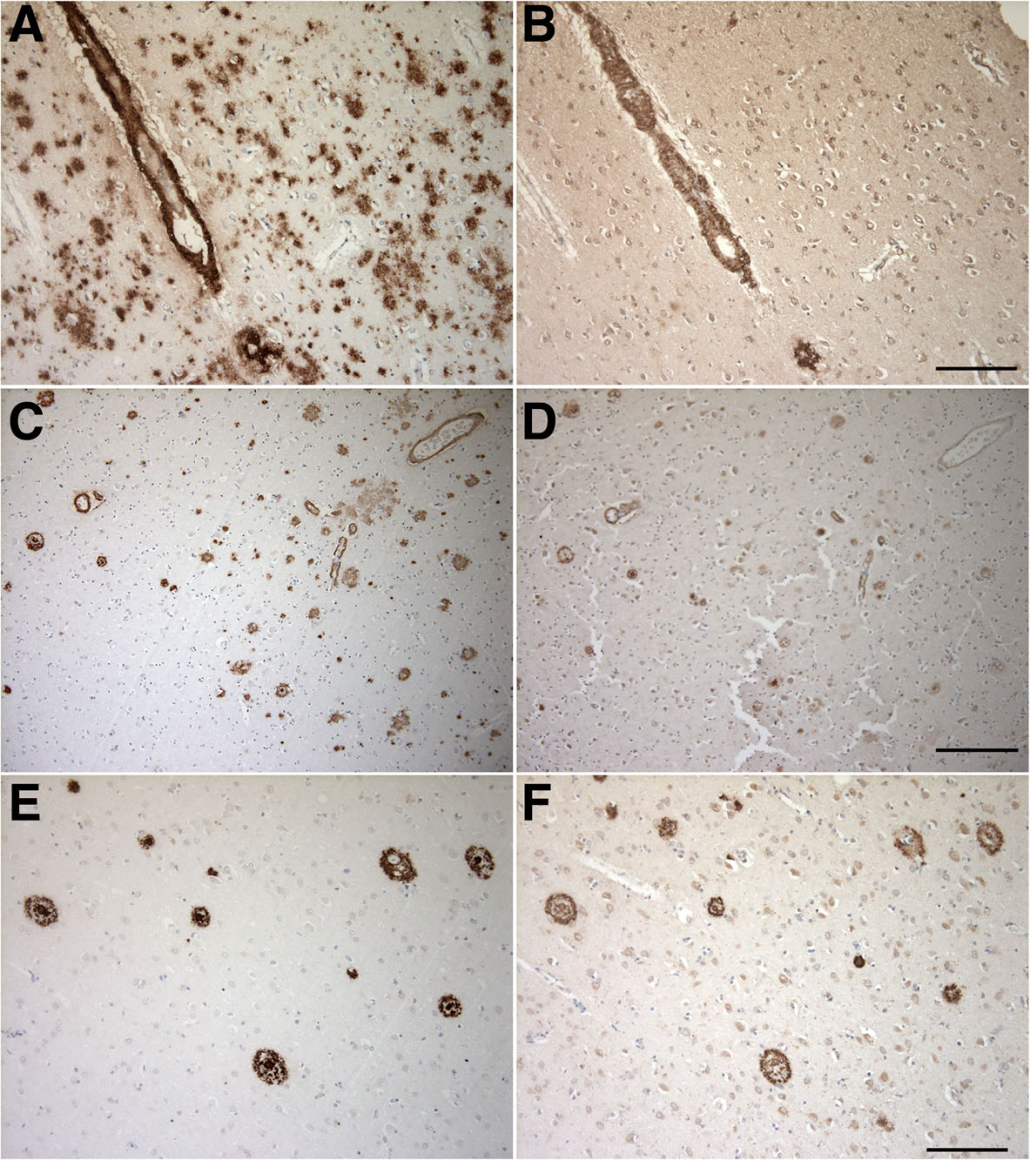
Immunohistochemical staining of amyloid-β (Aβ)_4–x_ peptides in sporadic Alzheimer’s disease (AD) cases. With IC16 recognizing full-length Aβ peptides, abundant extracellular (**a**, **c**, **e**) and vascular (**a**, **c**) amyloid deposits were detected in sporadic AD brains (**a**). In contrast, after staining with the Aβ_4–x_ specific 029-2 antibody, immunoreactivity was restricted to blood vessels (**b**, **d**) and neuritic plaques (**d**, **f**), while diffuse amyloid deposits were completely negative (**b**). Scale bar = 100 μm (**a**, **b**, **e**, **f**); 200 μm (**c**, **d**)

**Fig. 4:**
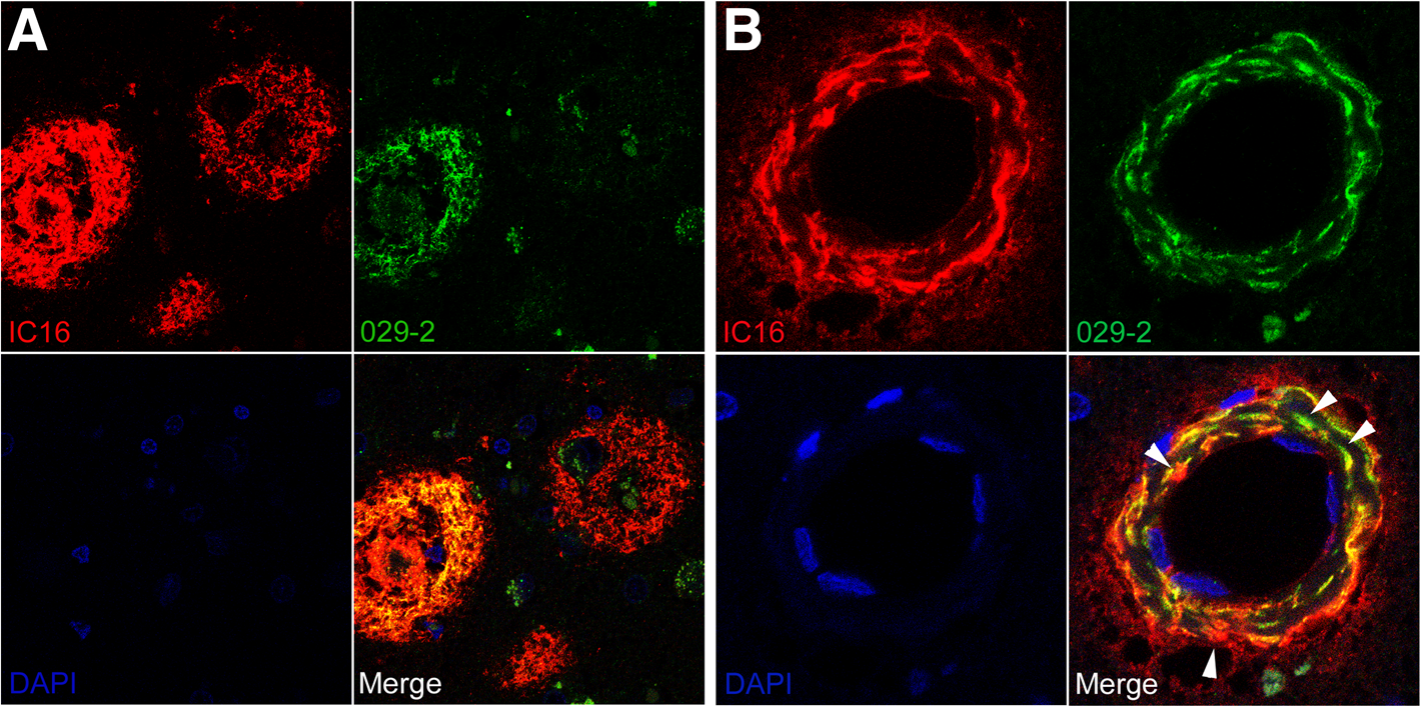
Fluorescent immunostaining in sporadic Alzheimer’s disease cases. Confocal microscopy using IC16 (*red*), 029-2 (*green*), and 4′,6-diamidino-2-phenylindole (DAPI; *blue*) reveals colocalization of IC16 and 029-2 staining in cored but not diffuse amyloid deposits (**a**). In blood vessels, partially overlapping staining profiles were observed; however, distinct immunoreactivity of IC16 and 029-2 also was evident within the vessel wall (**b**, *arrowheads*). Scale bar = 33 μm (**a**); 15 μm (**b**)

On tissue slides from human patients with sporadic AD, 029-2 labeled extracellular amyloid deposits as well as blood vessels (Fig. 3). In contrast to IC16, which detects full-length Aβ peptides and N-truncated variants and decorates either cored plaques or more diffuse extracellular Aβ deposits (Fig. 3a, c, e), 029-2 staining was restricted mainly to compact neuritic plaques (Fig. 3f) and plaque cores (Additional file 2) as well as vascular deposits (Fig. 3b, d). Table 1 summarizes a semiquantitative analysis of 029-2 versus IC16 staining in sporadic AD and nondemented control specimens, in which the immunoreactivity in the form of cerebral amyloid angiopathy (CAA) and extracellular amyloid deposition was assessed. Double-labeling and confocal microscopy using IC16 (red) and 029-2 (green) confirmed the presence of Aβ_4–x_ peptides predominantly in cored neuritic plaques, whereas more diffuse extracellular deposits showed only IC16 immunoreactivity (Fig. 4a). Confocal analysis of blood vessels revealed partial colocalization of Aβ_4–x_ peptides with IC16 immunoreactivity in the vessel wall. However, in a minor subset of vessels, the vessel walls showed either IC16 or 029-2 immunoreactivity and a nonoverlapping staining pattern (Fig. 4b, arrowheads).

### Aβ_4–x_ in transgenic mouse models

**Fig. 5:**
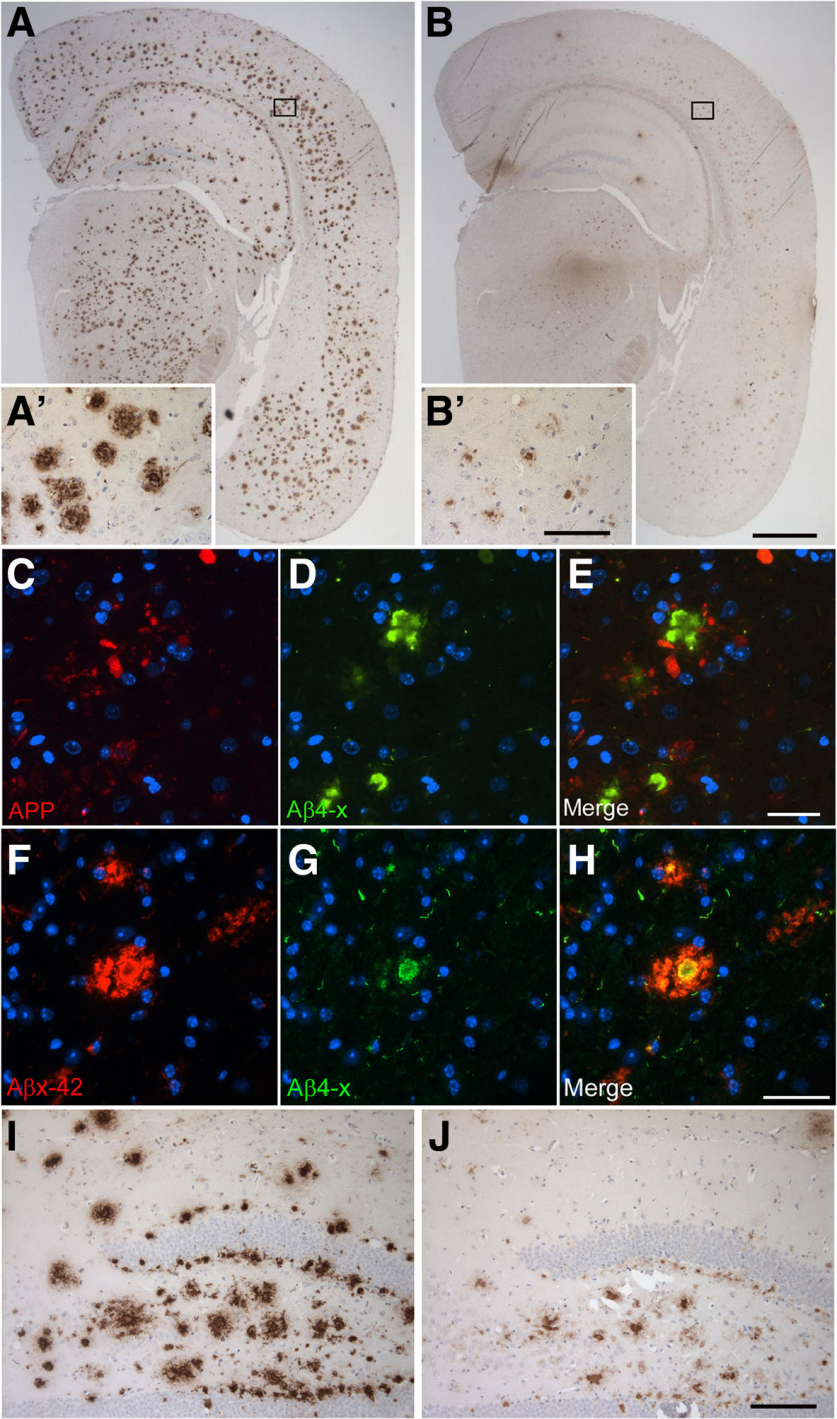
Amyloid-β (Aβ)_4–x_ peptides in transgenic Alzheimer’s disease (AD) mouse models. Using antibody D3E10 against Aβ_42_, abundant extracellular deposits were detected in 10-month-old 5XFAD mice by immunohistochemistry (**a**, **a′**). In contrast, the Aβ_4–x_-specific antibody 029-2 showed a strongly reduced staining pattern that was restricted to amyloid cores (**b**, **b′**). Staining with an amyloid precursor protein (APP) antibody decorated only dystrophic neurites (**c**) surrounding the Aβ_4–x_-positive plaque core (**d**, **e**). The restricted Aβ_4–x_ immunoreactivity within plaque cores was further demonstrated using double-labeling of D3E10 (**f**) and 029-2, which decorated only the central plaque core (**g**, **h**). A similar staining pattern to that in 5XFAD mice was observed in 8-month-old APP/PS1KI mice, as exemplified in the hippocampus (D3E10 [**i**] and 029-2 [**j**]). Scale bar = 1000 μm (**a**, **b**); 60 μm (**a′**, **b′**, **f**–**h**); 33 μm (**c**–**e**); 100 μm (**i**, **j**)

**Fig. 6:**
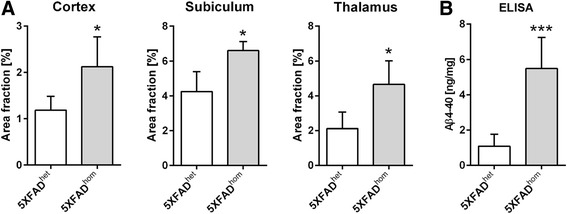
Increased amyloid-β (Aβ)_4–x_ levels in homozygous 5XFAD mice **a**. Quantification of extracellular 029-2-positive plaque load in cortex, subiculum, and thalamus in heterozygous and homozygous 9-month-old 5XFAD mice. **b** Quantification of sodium dodecyl sulfate-soluble Aβ_4–40_ levels in brains of heterozygous and homozygous 5-month-old 5XFAD mice revealed a significant gene dose-dependent increase. ***p < 0.001; *p < 0.05. *ELISA* Enzyme-linked immunosorbent assay, *FAD* Familial Alzheimer’s disease

With antibody 029-2 against Aβ_4–x_, abundant extracellular amyloid plaques were detectable in 10-month-old 5XFAD (Fig. 5b) and 8-month-old APP/PS1KI mice (Fig. 5j). Compared with staining with the Aβ_42_-selective antibody D3E10 (Fig. 5a), 029-2 immunoreactivity was restricted to the plaque core (Fig. 5b) in aged 5XFAD mice. Double-labeling with an APP antibody (red) confirmed the localization of Aβ_4–x_ (green) in the plaque core, whereas APP was detected largely within dystrophic neurites in the vicinity of the amyloid plaque (Fig. 5c–e). This was further supported by double-labeling with D3E10 and 029-2, clearly indicating that Aβ_4–x_ immunoreactivity was restricted to the central plaque core (Fig. 5f–h). Similar results were obtained for the APP/PS1KI line, in which D3E10 displayed a more diffuse and dissipated staining pattern (Fig. 5i), whereas 029-2 labeled only the central plaque core (Fig. 5j). A quantitative analysis of extracellular Aβ_4–x_-positive deposits using antibodies 029-1 and 029-2 was carried out in the cortex, subiculum, and thalamus of 9-month-old heterozygous and homozygous 5XFAD mice (Fig. 6a and Additional file 3). For both antibodies, a significantly increased plaque load could be detected in all analyzed areas in homozygous compared with heterozygous 5XFAD mice (all *p* < 0.05). In addition, to evaluate the suitability of the 029-2 antibody for ELISA quantification, Aβ_4–40_ levels were determined in the SDS-soluble brain fraction of 5-month-old heterozygous and homozygous 5XFAD mice. As expected, significantly increased Aβ_4–40_ levels were detected in homozygous 5XFAD mice (*p* < 0.001), supporting a gene dose-dependent increase in insoluble Aβ_4–40_ peptide levels (Fig. 6b).

## Discussion

In addition to full-length Aβ peptides starting with an aspartate in position 1 (Aβ_1–x_), a variety of N-terminally truncated and posttranslationally modified Aβ peptides have been detected in human AD brains [14, 16, 17]. N-terminal truncations were shown to promote the aggregation propensity of Aβ peptides [18]. However, the presence of N-truncated peptides has been demonstrated mainly by mass spectrometry following immunoprecipitation with generic Aβ antibodies such as 4G8 or 6E10 [14, 15, 21], and their genesis in vivo is mostly unresolved. Consequently, the functional role of N-truncated Aβ peptides in the pathogenesis of AD has remained unclear, including fundamental questions about the abundance and distribution of N-truncated isoforms. This shortcoming has been particularly obvious for the Aβ_4–x_ peptides starting with Phe in position 4, which have been proposed to be an abundant Aβ species in AD brains but for which no specific antibodies have been generated to date. We have previously reported a monoclonal antibody, NT4X-167, that preferentially detected Aβ_4–x_ peptides and protected primary cortical neurons from the toxicity of Aβ_4–42_ peptides in vitro [30]. However, in addition to monomeric and oligomeric Aβ_4–x_ peptides, NT4X-167 was shown to recognize Aβ_pE3–x_ peptides. Hence, this antibody is not suitable for accurate measurement of the abundance and distribution of Aβ_4–x_ peptides. We have now raised polyclonal antibodies by immunizing guinea pigs with the six-amino acid peptide (FRHDSG) corresponding to residues 4–9 of the Aβ peptide sequence. The specificity of these antibodies for Aβ_4–x_ peptides was confirmed by CIEF immunoassay and urea SDS-PAGE, and no cross-reactivity for Aβ_1–40_, Aβ_2–40_, Aβ_3–40_, Aβ_pE3–40_, and Aβ_5–40_ was observed. Furthermore, in immunohistochemical staining, the immunoreactivity could be entirely blocked by preabsorption with Aβ_4–40_ but not Aβ_1–40_ peptides. Two independent animals were immunized and yielded two antisera, 029-1 and 029-2, with nearly identical immunoreactivity, indicating that Aβ_4–9_ might be a reliable immunogen to raise Aβ_4–x_-specific antibodies in guinea pigs. Compared with the well-established antibody IC16, which preferentially detects full-length Aβ peptides, the immunohistochemical staining patterns of the newly generated Aβ_4–x_-specific antibodies were quantitatively and qualitatively different. In brain sections of both patients with sporadic AD and two AD mouse models, the distribution of Aβ_4–x_ peptides was restricted largely to amyloid plaque cores and CAA, whereas diffuse amyloid deposits were negative. The presence of Aβ_4–x_ peptides in amyloid plaque cores raises the question whether these truncated species are critical in the very early stages of the pathology. We have not yet conducted a comprehensive longitudinal study comparing different animal ages and time points before or after the onset of amyloid deposition. However, using two-dimensional Western blotting combined with mass spectrometry, N-terminally truncated Aβ peptide species starting at position 4 or 5 have already been detected at 2.5 months of age in the APP/PS1KI line, indeed indicating a very early appearance of these truncated species [26]. In good agreement and using a similar experimental approach, Sergeant and colleagues reported that Aβ aggregates at the first stages of amyloid deposition in nondemented individuals with amyloid and tau pathologies are composed predominantly of N-truncated variants, including Aβ_4–x_ peptides [31]. Antibodies raised in guinea pigs are especially useful for colocalization studies because most high-quality antibodies against other Aβ species or APP fragments have previously been generated in either mice or rabbits. Indeed, double-immunofluorescence staining demonstrated Aβ_4–x_-positive amyloid plaque cores decorated by APP-positive dystrophic neurites with no overlap in the fluorescent signals. In line with this observation, no intraneuronal staining was observed for Aβ_4–x_ peptides in mice at the ages of 8–10 months. However, it could be worth studying younger animals because intraneuronal Aβ accumulation is most prominent in young mice prior to amyloid plaque formation [32]. Overall, the distribution of Aβ_4–x_ peptides was also substantially different from the staining pattern reported for other N-truncated species, including Aβ_2–x_ [7] or Aβ_5–x_ [10, 11], which were not or less confined to cored neuritic plaques. Previous studies have demonstrated that Aβ_4–x_ peptides rapidly formed soluble oligomers and fibrillar higher-molecular-weight aggregates [33]. This biochemical property might explain not only the confined localization of Aβ_4–x_ peptides to amyloid cores but also their high neurotoxicity in vitro and in vivo. Short-term exposure of primary cortical neuron cultures to Aβ_4–40_ and Aβ_4–42_ peptides resulted in a concentration-dependent cytotoxic effect with comparable effect sizes to Aβ_1–42_. Furthermore, the expression of Aβ_4–42_ under the control of a neuronal promotor caused age-dependent behavioral deficits and hippocampal neuron loss in a transgenic mouse model (Tg4-42) [33].

Another important unresolved issue is the abundance of Aβ_4–x_ peptides in relation to full-length Aβ peptides in both AD and transgenic mouse models of the disease. To start to address this issue and to evaluate the novel Aβ_4–x_ antibodies for quantitative analysis, we combined the 029-2 antibody with a C-terminus-specific Aβ_40_ antibody [29] in a sandwich ELISA. In the SDS-soluble brain fraction of 5-month-old heterozygous 5XFAD mice, this assay detected around 1 ng of Aβ_4–40_ per milligram of tissue with approximately fivefold higher levels in homozygous 5xFAD mice of the same age. In a previous study, we had determined the levels of full-length Aβ_1–40_ and Aβ_1–42_ peptides in the same brain extracts of the same animal cohort with a comparable ELISA system and IC16 as a capture antibody [28]. Combining the results from both studies indicates that Aβ_1–40_ and Aβ_1–42_ peptides are approximately 75-fold and 200-fold more abundant, respectively, than Aβ_4–40_ peptides in the 5XFAD mouse model at 5 months of age. This also fits with a matrix-assisted laser desorption/ionization time-of-flight mass spectrometric analysis of formic acid brain extracts of 7-month-old 5XFAD mice. Although peptide concentrations cannot be deduced from peak heights in mass spectra, this analysis showed that the signal intensities generated by the Aβ_4–40_ and Aβ_4–42_ peptides were only a small fraction of the Aβ_1–40_ and Aβ_1–42_ peaks [5]. Taken together, these results indicate that, quantitatively, N-truncated Aβ_4–x_ peptides are a minor Aβ species in the 5XFAD mouse model. However, evidence from human studies suggests that the proportions of N-truncated to full-length Aβ peptides might be substantially different in the brains of patients with AD. Already more than 30 years ago, it was shown by N-terminal sequencing analysis of Aβ peptides purified from amyloid plaque cores that only around 10% of peptides displayed an intact N-terminus, whereas > 60% started with the Phe residue in position 4 [19]. Another sequencing study confirmed that Aβ peptides starting with Phe represented the major component of plaques, whereas full-length Aβ starting with Asp was detected predominantly in the vasculature [20]. In addition, later studies using mass spectrometry have generally supported that Aβ_1–42_, Aβ_pE3–42_, and Aβ_4–42_ belong to the Aβ peptide species with a high prevalence in AD brains [14, 15, 21, 34]. Finally, comparative biochemical studies of amyloid plaques isolated from human AD brains and APP transgenic mouse models have also shown that N-truncated Aβ peptides are much more prevalent in patients with AD, and it has been proposed that the greater abundance of N-truncated Aβ peptides is at least in part responsible for the substantially lower solubility of amyloid plaque material from humans [35, 36]. In any case, additional studies in human AD brains using genuinely quantitative methods to determine the abundance of Aβ_4–x_ peptides and their distribution in soluble and insoluble brain fractions are clearly warranted. How Aβ_4–x_ peptides are generated remains entirely unclear. Although there is evidence that enzymatic activities can facilitate N-terminal Aβ truncations such as Aβ_2–x_ [37, 38], no enzymes have yet been identified that are able to generate Aβ_4–x_ peptides. Nonenzymatic generation of truncated Aβ peptides has also been proposed, and it has been shown that full-length Aβ peptides can spontaneously decompose into shorter N- and C-terminally truncated isoforms in vitro in the absence of proteases [39, 40]. With respect to N-terminal truncations, spontaneous decomposition of full-length to Aβ_3–x_ peptides with subsequent cyclization of the N-terminal Glu to pGlu has been demonstrated, but there is only very limited evidence that a similar process could produce Aβ_4–x_ peptides [39]. Our finding that Aβ_4–x_ peptides are confined largely to amyloid cores supports an important role of these N-truncated Aβ species in the process of amyloid plaque formation. Beyond that, further understanding of the pathological relevance of Aβ_4–x_ peptides will likely require clarification of their origin and the subsequent generation of genetic loss-of-function models.

## Conclusions

Antibodies developed in the present study selectively detect Aβ_4–x_ species and might represent useful research tools for immunohistochemical or biochemical analyses of the occurrence or distribution of these peptides. Our analysis reveals that Aβ_4–x_ is restricted mainly to amyloid plaque cores and CAA in AD and DS cases, as well as in the 5XFAD and APP/PS1KI transgenic mouse lines of AD. This is in line with previous reports demonstrating the high aggregation propensity of these N-terminally truncated Aβ variants.

## Additional files

Additional file 1: Figure S1. CIEF electropherograms for antibodies 6E10 and 029-1. (PDF 184 kb)

Additional file 2: Figure S2. Quantification of extracellular 029-1 positive plaque load in cortex, subiculum, and thalamus in heterozygous and homozygous 9-month-old 5XFAD mice. (PDF 119 kb)

Additional file 3: Figure S3. Predominant plaque core staining using 029-2 in a patient with sporadic AD. (PDF 183 kb)

## Abbreviations

Aβ: Amyloid-β
AD: Alzheimer’s disease
ApoE: Apolipoprotein E
APP: Amyloid precursor protein
BSA: Bovine serum albumin
CAA: Cerebral amyloid angiopathy
CIEF: Capillary isoelectric focusing
DAB: Diaminobenzidine
DAPI: 4′,6-Diamidino-2-phenylindole
DMSO: Dimethyl sulfoxide
DS: Down syndrome
ECL: Electrochemiluminescence
ELISA: Enzyme-linked immunosorbent assay
FAD: Familial Alzheimer’s disease
IgG: Immunoglobulin G
KLH: Keyhole limpet hemocyanin
NDC: Nondemented control subject
PBS-T: Phosphate-buffered saline with 0.1% Tween 20
Phe: Phenylalanine
PSEN1: Presenilin-1
PSL: Peptide Specialty Laboratory
RT: Room temperature
SDS: Sodium dodecyl sulfate
SP: Senile plaques
