# Supplementary material for: N-truncated Aβ4–x peptides in sporadic Alzheimer’s disease cases and transgenic Alzheimer mouse models
Source: Alzheimers Res Ther. 2017 Oct 4;9:80. doi: 10.1186/s13195-017-0309-z (PMC5628465; doi:10.1186/s13195-017-0309-z)

Supplementary information:

Additional File 1: Figure S1

Antibody 029-1 showed high selectivity for Aβ peptides starting with the Phe residue in position 4. Synthetic Aβ peptides with different N-termini were analyzed by capillary isoelectric focusing immunoassay. Mixtures of $Aβ_{1\text{-}40}$, $Aβ_{2\text{-}40}$ and $Aβ_{5\text{-}40}$ (top electropherogram), $Aβ_{3\text{-}40}$ (second electropherogram), $Aβ_{pE3\text{-}40}$ (third electropherogram) and $Aβ_{4\text{-}40}$ (bottom electropherogram) were subjected to isoelectric focusing in microcapillaries and probed with antibodies 6E10 (**A**) or 029-1 (**B**).

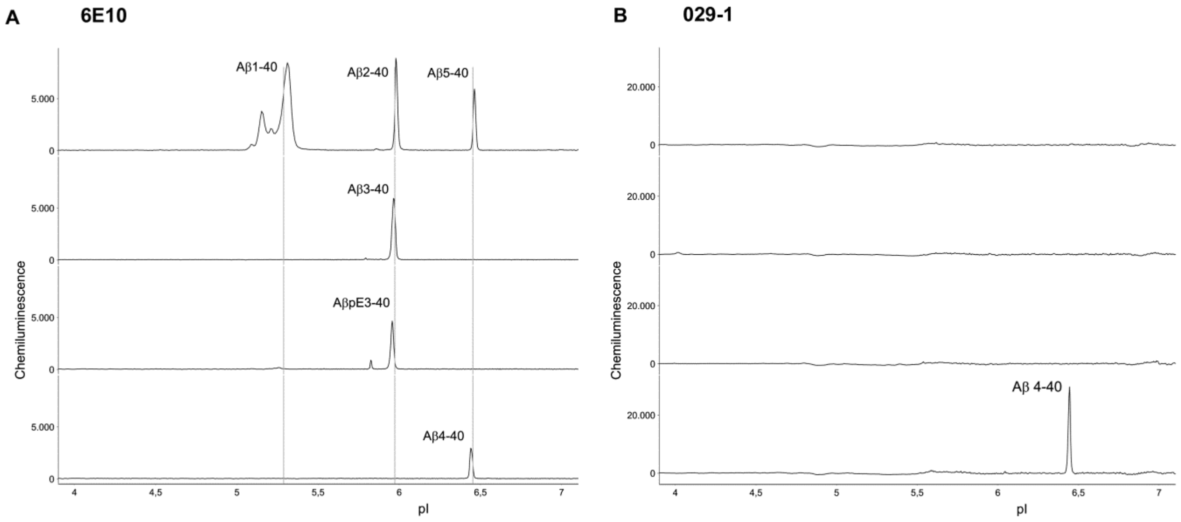

Supplement: Supplementary file 1 — CIEF electropherograms for antibodies 6E10 and 029-1. (PDF 184 kb) [file 13195_2017_309_MOESM1_ESM.pdf]
