# Supplementary material for: N-truncated Aβ4–x peptides in sporadic Alzheimer’s disease cases and transgenic Alzheimer mouse models
Source: Alzheimers Res Ther. 2017 Oct 4;9:80. doi: 10.1186/s13195-017-0309-z (PMC5628465; doi:10.1186/s13195-017-0309-z)

Additional File 2: Figure S2

Quantification of extracellular 029-1 positive plaque load in cortex, subiculum and thalamus in heterozygous and homozygous 9-month-old 5XFAD mice.

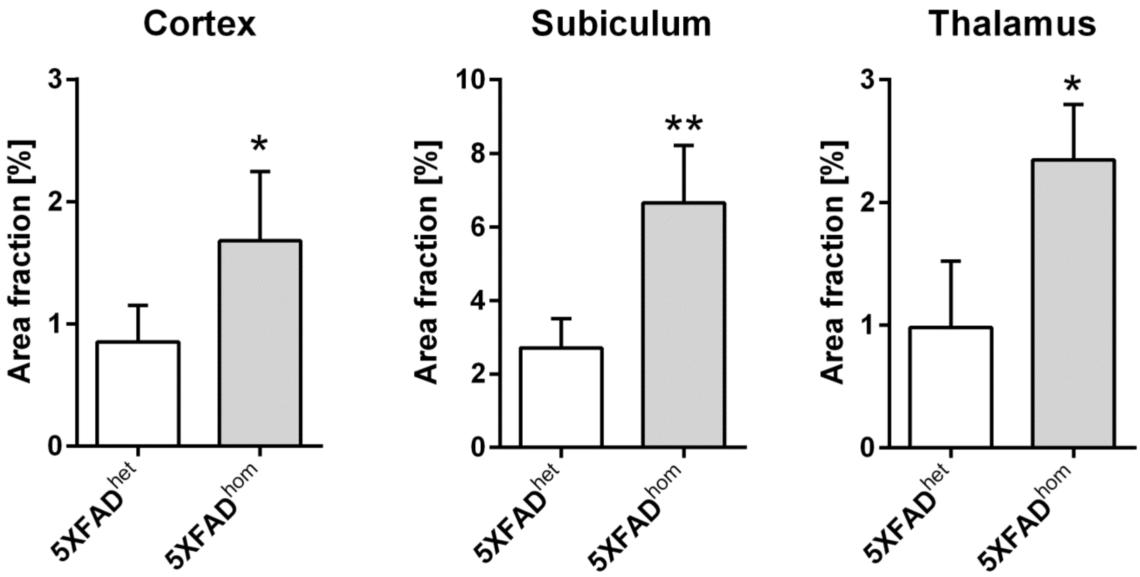

Supplement: Supplementary file 2 — Quantification of extracellular 029-1 positive plaque load in cortex, subiculum, and thalamus in heterozygous and homozygous 9-month-old 5XFAD mice. (PDF 119 kb) [file 13195_2017_309_MOESM2_ESM.pdf]
