# Supplementary material for: N-truncated Aβ4–x peptides in sporadic Alzheimer’s disease cases and transgenic Alzheimer mouse models
Source: Alzheimers Res Ther. 2017 Oct 4;9:80. doi: 10.1186/s13195-017-0309-z (PMC5628465; doi:10.1186/s13195-017-0309-z)

Additional File 3: Figure S3

Example showing predominant labelling of the plaque core using 029-2 (purple) in a sporadic AD patient while IC16 labels also more diffuse portions of amyloid plaques (green). As secondary antibodies, donkey-anti-mouse DyLight488 (green, Thermo Fisher Scientific) and goat-anti-guinea pig DyLight650 (purple, Thermo Fisher Scientific) were used. Scale bar: 33 µm

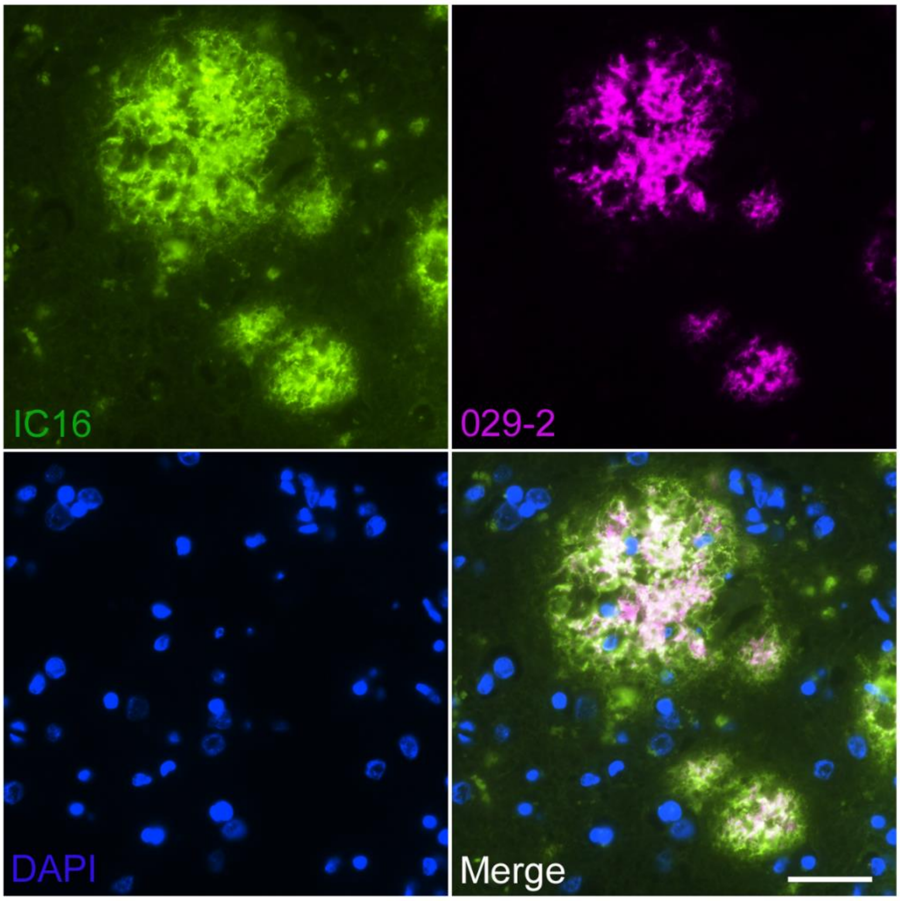

Supplement: Supplementary file 3 — Predominant plaque core staining using 029-2 in a patient with sporadic AD. (PDF 183 kb) [file 13195_2017_309_MOESM3_ESM.pdf]
